# Clinical Features of Multidrug-Resistant Gram-Negative Bacteremia: A Comparative Study of Cancer and Non-Cancer Patients

**DOI:** 10.3390/microorganisms13092110

**Published:** 2025-09-10

**Authors:** Destyn Dicharry, Deborah G. Smith, Muhammad H. Khan, Michelle Self, Cameron Parikh, Alexandre E. Malek

**Affiliations:** 1 Division of Infectious Diseases, Department of Medicine, School of Medicine, LSU Health Shreveport, Shreveport, LA 71103, USA; dld006@lsuhs.edu (D.D.); muhammad.khan03@lsuhs.edu (M.H.K.); cameronparikh15@gmail.com (C.P.); 2 Department of Public Health, LSU Health Shreveport, Shreveport, LA 71103, USA; deborah.smith@lsuhs.edu; 3 Division of Palliative Care, Department of Medicine, LSU Health Shreveport, Shreveport, LA 71103, USA; michelle.self@lsuhs.edu

**Keywords:** multidrug-resistant Gram-negative bacteria, bacteremia, bloodstream infection, cancer patients

## Abstract

Multidrug-resistant Gram-negative bacteremia (MDR-GNB) is a significant health threat associated with increased morbidity and mortality rates. Patients with cancer are particularly vulnerable to MDR-GNB due to immunosuppression and frequent healthcare exposure. The aim of this study was to evaluate risk factors, 30-day mortality, and outcomes in cancer and non-cancer patients. We conducted a retrospective study of adult patients aged 18 years or older with MDR-GNB who were hospitalized at Ochsner LSU Health—Academic Medical Center between January 2018 and July 2022. We collected data about demographics, comorbidities, cancer diagnosis, causative organisms, infection source, antibiotic therapy, and clinical outcomes. A total of 112 patients with MDR-GNB were included, where 31 patients (27.7%) had cancer and 81 patients (72.3%) did not. Cancer patients were more frequently male and white (74.2% vs. 58.0%, *p* = 0.114 and 45.2% vs. 25.9%, *p* = 0.031). Diabetes mellitus was more common in non-cancer patients, but it was associated with increased mortality risk in the cancer group (OR = 2.39, 95% CI: 1.125–5.074). Enterobacteriaceae species were the most frequently isolated organisms (83.0%), with no significant difference between groups. The most common source of infection was genitourinary (49.1%). ICU admission was more frequent in non-cancer patients (49.4% vs. 25.8%, *p* = 0.024). However, cancer patients had a higher ICU admission mortality risk (OR 2.156, 95% CI: 1.058–4.395) and recent hospitalization rates (67.7% vs. 40.7%, *p* = 0.011), both associated with increased mortality risk. Cancer patients had a significantly higher 30-day mortality rate (39.0% vs. 16.4%, *p* = 0.017; OR = 3.012, 95% CI: 1.190–7.622) and hospice admissions (22.6% vs. 3.7%, *p* = 0.002; OR = 7.583, 95% CI: 1.819–31.618). These findings emphasize the urgent need for early microbiological identification, targeted antimicrobial therapy, and improved infection control strategies. Given the rising prevalence of MDR-GNB pathogens, future research is needed for prompt appropriate antibacterial therapy based on risk stratification and enhanced antimicrobial stewardship programs as these are critical for high-risk cancer patients.

## 1. Introduction

Multidrug-resistant Gram-negative bacteremia (MDR-GNB) is an emerging global health concern associated with significant morbidity and mortality. The increased rate of antimicrobial resistance, particularly in Gram-negative bacteria, has rendered the management of bloodstream infections (BSIs) more challenging and led to limited treatment options [1]. The increasing prevalence of extended-spectrum β-lactamase (ESBL)-producing and carbapenem-resistant *Enterobacteriaceae (CRE)*, as well as quinolone- and carbapenem-resistant *Pseudomonas aeruginosa* and *Acinetobacter baumannii* organisms (CRO), presents a growing challenge for clinicians in tackling MDR-GNB [2].

Previous studies suggest that cancer patients with MDR-GNB have worrisome outcomes, including prolonged hospital stays, higher intensive care unit (ICU) admission rates, and increased mortality [3]. However, data directly comparing the clinical characteristics and outcomes of MDR-GNB in cancer and non-cancer patients remain limited in the literature, and there is very little data about the burden of bloodstream infection secondary to resistant bacteria comparing both groups of patients. This study aims to compare the clinical characteristics, microbiological profiles, infection sources, treatment approaches, and outcomes of MDR-GNB in cancer and non-cancer patients to identify key differences and inform strategies for improving outcomes in high-risk populations.

## 2. Methods

### 2.1. Study Design and Setting

This retrospective cohort study examined the clinical characteristics and 30-day mortality outcomes of patients with bacteremia caused by multidrug-resistant Gram-negative bacteria. This study was conducted at Ochsner LSU Health, Shreveport—Academic Medical Center and included cases identified between January 2018 and July 2022. This hospital is a multispecialty and acute care facility that also houses North Louisiana’s Level 1 trauma center and the region’s burn unit and stroke center. The hospital serves as a major referral center for critical and specialized care in the region.

### 2.2. Study Population

We included 112 adult patients (aged 18 years or older) with confirmed multidrug-resistant Gram-negative bacteremia, as identified from microbiology laboratory records. Patients were stratified into two groups based on cancer status: those with a documented cancer diagnosis (*n* = 31) and those without cancer (*n* = 81). Inclusion criteria included a positive blood culture for a multidrug-resistant Gram-negative organism and complete clinical data. Patients with polymicrobial bacteremia or incomplete records were excluded.

### 2.3. Data Collection

Data were extracted from electronic medical records and included demographic characteristics (age, sex, race/ethnicity), comorbid conditions (e.g., diabetes mellitus), prior healthcare exposures (e.g., hospitalization in the previous 3 months), ICU admissions, clinical presentation, microbiological results, timing and appropriateness of antimicrobial therapy, ID consultations, and 30-day mortality outcomes. Predicted probabilities of 30-day mortality were generated using logistic regression.

### 2.4. Statistical Analysis

Data were analyzed using IBM SPSS Statistics v29. Continuous variables were summarized as means ± SD or medians with interquartile ranges, and categorical variables were summarized as frequencies and percentages. Bivariate comparisons between cancer and non-cancer patients used Pearson’s χ^2^ or Fisher’s exact test for categorical variables and independent-samples *t*-tests for continuous variables. A multivariable logistic regression model was used to examine predictors of 30-day mortality. Covariates included cancer status, sex, diabetes mellitus, ICU admission, recent hospitalization within the last 3 months, ethnicity, and age (categorized as <40 vs. ≥40 years). Variables were coded as binary (0 = reference; 1 = risk category), and the first category was set as the reference level. Adjusted odds ratios (aORs) with 95% confidence intervals (CIs) were calculated. To visually examine risk stratification by cancer status, predicted probabilities from the logistic regression model were saved and plotted using a boxplot, with predicted 30-day mortality on the Y-axis and cancer status on the X-axis. Group medians were compared using the Mann–Whitney U test. Model fit was assessed using the Hosmer–Lemeshow goodness-of-fit test, with *p* > 0.05 indicating good calibration. Two-sided *p* < 0.05 was considered statistically significant.

### 2.5. Microbiological Identification and Susceptibility Testing

Microbiological identification in this study focused on multidrug-resistant Gram-negative organisms, including carbapenem-resistant organisms (CROs) and extended-spectrum β-lactamase (ESBL) producers. CROs were detected from routine clinical and surveillance samples (e.g., rectal swabs, fecal specimens, wound swabs, urine), which were cultured in the hospital laboratory with results typically available within one to three days. CROs were defined as carbapenem-resistant organisms including carbapenemase-producing bacteria capable of hydrolyzing carbapenem antibiotics. ESBL-producing organisms were identified through culture-based susceptibility testing, with reduced susceptibility to third-generation cephalosporins prompting confirmatory testing in line with established laboratory practice [4]. ESBLs were defined as β-lactamases that confer resistance to penicillins, cephalosporins, and aztreonam but remain inhibited by β-lactamase inhibitors. Antibiotic susceptibility testing for both CRO and ESBL isolates was performed to guide treatment, and resistance results were reported in accordance with institutional protocols and standard guidelines.

### 2.6. Ethical Considerations

This study was approved by the Institutional Review Board of Louisiana State University Health, Shreveport, and the study was performed in line with the principles of the Declaration of Helsinki. The requirement for informed consent was waived due to the retrospective nature of the study and the use of de-identified patient data.

## 3. Results

A total of 112 patients with bacteremia secondary to multidrug-resistant Gram-negative bacteria met the inclusion criteria and were included in the analysis. Among them, 31 patients (27.7%) had cancer and 81 (72.3%) did not. Overall, 42 (37.5%) patients were female and 70 (62.5%) were male. Patients with cancer were more frequently male (74.2%) compared to those without cancer (58.0%, *p* = 0.114) (Table 1). The mean age of patients in the cancer group was 67 years, compared to 55 years in the non-cancer group (*p*-value < 0.001). Regarding race and ethnicity, African American patients comprised the majority of the current cohort (66.1%). The proportion of White patients was significantly higher in the cancer group compared to the non-cancer group (45.2% vs. 25.9%, *p* = 0.031) (Table 1).

In terms of comorbidities, diabetes mellitus was significantly more common among non-cancer patients (48.1% vs. 22.6%, *p* = 0.014). However, in cancer patients, diabetes was associated with an increased risk of mortality with an odds ratio of 2.39 (95% CI: 1.125–5.074). Stroke history (22.2%, *p* = 0.004) and end-stage renal disease (13.6%, *p* = 0.031) were observed more frequently in the non-cancer group. Other comorbidities, including hypertension, HIV, COPD, CAD/CHF, and a recent history of COVID-19 infection, were comparable between both groups. (Table 1)

The most frequently isolated organisms were *Enterobacteriaceae* species, found in 83.0% of all cases, with no significant difference between cancer and non-cancer patients (83.9% vs. 82.7%, *p* = 0.884). Quinolone resistance was observed in 74.2% of isolates from cancer patients and 63.0% from non-cancer patients, with no statistical difference (*p* = 0.261). The most common source of infection was genitourinary (49.1%), followed by pulmonary (11.6%), intra-abdominal (2.7%), and skin and soft tissue infections (4.5%), with no significant differences in source distribution between groups (Table 2)

The majority of patients in both groups received empiric therapy at the time of signs and symptoms of infection and within 1 to 3 days of positive blood culture, with initiation on day 1 in 33.3% of cancer patients and 44.2% of non-cancer patients, and between days 2 and 3 in 51.9% of cancer patients and 27.3% of non-cancer patients (*p* = 0.169) (Table 2). While 12.9% of cancer patients and 4.9% of non-cancer patients were continued on empiric IV antibiotic therapy only, targeted IV antibiotic therapy was initiated in 92.9% of cases, with no significant difference between groups (87.1% in cancer patients vs. 95.1% in non-cancer patients, *p* = 0.143). The time to initiation of targeted therapy also did not differ significantly, with most patients receiving targeted therapy between 1 and 3 days after culture positivity.

The ICU admission was significantly more common in non-cancer patients (49.4%) compared to cancer patients (25.8%) (*p* = 0.024). The ICU admission rate among cancer patients was associated with increased mortality risk (OR = 2.156, 95% CI: 1.058–4.395) (Table 3A,B). A recent hospitalization within the past 3 months was also more frequent among cancer patients (67.7% vs. 40.7%, *p* = 0.011; OR = 3.055, 95% CI: 1.275–7.319). Laboratory findings showed that leukocytosis (WBC > 12,000) was more frequent in non-cancer patients (65.4% vs. 45.2%, *p* = 0.050), whereas thrombocytopenia (platelets < 150,000) was more common in cancer patients (48.4% vs. 31.6%, *p* = 0.101) (Table 2).

In terms of clinical outcomes, the 30-day mortality rate was significantly higher in cancer patients (39.0%) compared to non-cancer patients 16.4% (*p* = 0.017), with an odds ratio of 3.012 (95% CI: 1.190–7.622). Additionally, hospice admission was significantly more highly reported in cancer patients (22.6% vs. 3.7%, *p* = 0.002; OR = 7.583, 95% CI: 1.819–31.618) (Table 3).

**Figure 1:**
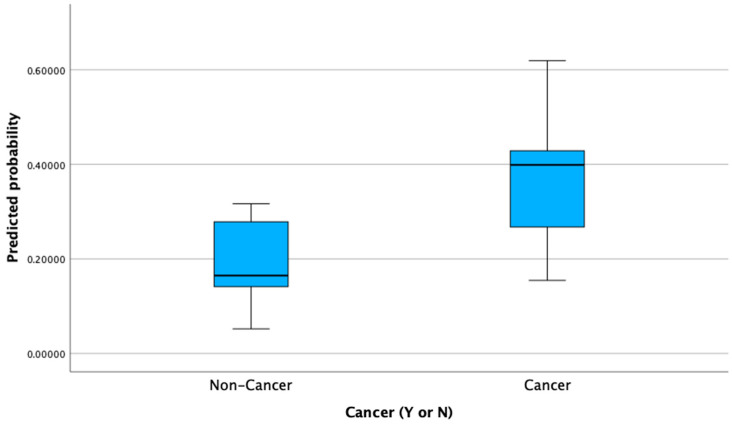
Predicted probability of 30-day mortality by cancer status.

In the multivariable logistic regression model, cancer status remained significantly associated with 30-day mortality after controlling for sex, age, diabetes, ICU admission, recent hospitalization, and ethnicity. Cancer patients had nearly four times the odds of death compared to non-cancer patients (aOR = 3.82, 95% CI: 1.23–11.86, *p* = 0.020). ICU admission was marginally associated with mortality (aOR = 2.35, *p* = 0.090), while other covariates were not statistically significant. The model demonstrated good calibration (Hosmer–Lemeshow χ^2^ = 0.34, *p* = 1.000) (Table 4). A boxplot of model-predicted mortality probabilities showed a significantly higher distribution among cancer patients compared to non-cancer patients (Mann–Whitney U = 253.0, Z = −6.522, *p* < 0.001; Figure 1).

A total of 112 multidrug-resistant Gram-negative isolates were identified, including 31 from cancer patients and 81 from non-cancer patients. The most frequent pathogen in both groups was ESBL-producing *Escherichia coli*, accounting for 61.3% of isolates in cancer patients and 50.6% in non-cancer patients. *Klebsiella pneumoniae* was the second most common organism (12.9% in cancer vs. 22.2% in non-cancer), with both ESBL-producing and carbapenem-resistant (CRO) strains identified. Other *Enterobacteriaceae* included *Klebsiella oxytoca* (ESBL and CRO), *Proteus mirabilis* (ESBL), and a single *Serratia marcescens* isolate (CRO, non-cancer group only). Non-fermenting Gram-negative bacilli were also recovered, including *Pseudomonas aeruginosa* (CRO, 9.7% in cancer vs. 8.6% in non-cancer) and *Acinetobacter baumannii*/*haemolyticus* (CRO, 9.7% in cancer vs. 9.9% in non-cancer). The full distribution of organisms stratified by cancer status is presented in Table 5.

## 4. Discussion

The emergence and increased prevalence of antimicrobial resistance (AMR) worldwide have created an unprecedented burden on healthcare settings and carried alarming outcomes on the most vulnerable patients [5]. Our findings align with other retrospective studies which identified *Enterobacteriaceae* as the most common pathogens in MDR-GNB cases and emphasized the high prevalence of quinolone resistance in cancer patients. Similar to our study; they reported that prior hospitalization was significantly associated with MDR-GNB infection, reinforcing on the risk of frequent contact to healthcare settings and the development of resistance [6]. In our study, we observed that recent hospitalization within the past three months was significantly more frequent among cancer patients (67.7% vs. 40.7%, *p* = 0.011) and was associated with increased risk of mortality (OR = 3.055, 95% CI: 1.275–7.319), highlighting the heightened risk of MDR-GNB acquisition and poor patient outcomes in this population. A recent study similarly reported that cancer patients with MDR-GNB had a high 30-day mortality along with increased neutropenia [5]. Their findings reinforce the need for early identification and aggressive infection control strategies to mitigate poor outcomes. Additionally, the study identified *Enterobacteriaceae* species as the predominant pathogens, with genitourinary infections being the most common source. This parallels our study’s results as we found *Enterobacteriaceae* in 83% of cases, with genitourinary infections being the most frequent source (49.1%) [5,6].

Our study, alongside the existing literature, emphasizes the urgent need for enhanced infection control measures and strengthened antimicrobial stewardship programs in hospitalized patients to avert the increased threat of AMR. Another study investigated the spectrum of bacteremia due to MDR-GNB in cancer patients and found that 13.7% of GNB cases in cancer patients were caused by multi-drug-resistant organisms [7]. Given the high mortality rates observed in cancer patients with bacteremia included in our study, early screening for MDR colonization, timely initiation of effective empiric therapy, and de-escalation strategies may be crucial for improving outcomes. This phenomenon has also been documented in the literature, as one study found higher ICU admission rates among cancer patients with MDR-GNB in addition to a documented higher mortality rate [7]. Our study reinforces these concerns, demonstrating a significantly higher 30-day mortality rate in cancer patients (39.0%) compared to non-cancer patients (16.4%, *p* = 0.017). Furthermore, ICU admission in our cohort was significantly more common in non-cancer patients (49.4% vs. 25.8%, *p* = 0.024), but ICU admission in cancer patients was associated with an increased mortality risk (OR = 2.156, 95% CI: 1.058–4.395). Another study investigated carbapenem-resistant *Pseudomonas aeruginosa* (CRPA) bloodstream infections in patients with hematologic malignancies and found a 37.5% 28-day mortality rate [8]. In addition, it was found that neutropenia, ICU admission, and high Pitt bacteremia scores are significant risk factors for mortality [8]. We similarly demonstrate the significant impact of MDR-GNB infections on ICU admissions, mortality risk, and 30-day mortality, reinforcing the need for proactive screening, timely treatment, and robust antimicrobial stewardship strategies.

The increased infection severity and mortality observed in cancer patients in our study and across the literature may be due to neutropenia, lymphocyte dysfunction, and invasive medical interventions [9]. A meta-analysis of *Stenotrophomonas maltophilia* bacteremia provides valuable insights into the key risk factors influencing patient mortality. The findings emphasize that immunosuppression, septic shock, and delayed or inappropriate antimicrobial therapy significantly worsen patient outcomes [10]. These results align with our broader understanding that timely and targeted treatment is crucial in managing multidrug-resistant infections, in addition to providing insight into the reasons that cancer patients have increased morbidity and mortality when fighting these resistant infections. A similar study investigating the risk factors and outcomes of bacteremia in cancer patients found that neutropenia and recent chemotherapy were associated with bacteremia in these patients [11], mirroring our finding that cancer patients were significantly more likely to be neutropenic (*p* = 0.050). Moreover, our study found that diabetes mellitus was significantly more common among non-cancer patients (48.1% vs. 22.6%, *p* = 0.014), yet in cancer patients, diabetes was associated with an increased risk of mortality (OR = 2.39, 95% CI: 1.125–5.074), emphasizing the potential compounding risk of metabolic disorders in this population. This is documented in previous studies as individuals with diabetes have been observed to be at an increased risk of bacteremia, which may be due to decreased immunity along with other comorbidities [12].

A longitudinal study investigating epidemiological trends in BSI causing microorganisms among patients with hematological malignancies between 2011 and 2021 found a gradual shift from Gram-positive to Gram-negative organisms [13]. This shift along with the increased antibiotic resistance and increased morbidity and mortality observed in our study, sheds light on the importance of focusing on MDR GNB patients in this population. The differing characteristics of MDR-GNB BSI in cancer and non-cancer patients provided by our study may provide important insights into how to improve patient outcomes.

The high prevalence of ESBL-producing *Enterobacteriaceae* suggests that carbapenem-sparing regimens (e.g., β-lactam/β-lactamase inhibitor combinations) should be explored to prevent further resistance [14]. Another study has also identified ESBL-producing *E. coli* as a predominant pathogen regarding MDR-GNB infections, finding that ESBL production was the predominant resistance mechanism most found in *E. coli* [7]. This emphasizes the clinical burden of B-lactam resistance in MDR-GNB infections. Our study corroborates these findings, identifying ESBL-producing *E. coli* and *Klebsiella* spp. as dominant MDR pathogens in both cancer and non-cancer patients.

One study highlights the increasing prevalence and clinical impact of multidrug-resistant organisms (MDROs) in hematological cancer patients with bloodstream infections (BSIs) over a 20-year period. The rate of MDRO-related BSIs rose fourfold, from 10.3% (2003–2007) to 39.7% (2018–2022) (*p* < 0.001), with Gram-negative MDROs increasing significantly. This study found that MDRO infections were associated with a significantly higher 30-day mortality rate (48.1% vs. 17.4%, *p* < 0.001), particularly in newly diagnosed and relapsed/refractory cancer patients. Carbapenem-resistant Gram-negative bacteria exhibited the highest mortality (61.9%), emphasizing their role as a major threat to immunocompromised patients [15]. This study, along with ours, underscores the need to prevent the spread of resistant strains to protect this vulnerable patient population.

A similar 25-year longitudinal study found the same to be true when they found a significant rise in MDR Gram-negative bacilli (GNB) infections, with ESBL-producing *E. coli* and *Klebsiella* spp. being the predominant pathogens. In addition, patients with MDR BSI had higher mortality rates (22.9% vs. 14%, *p* < 0.001) [16]. These findings align with our study, which similarly identified ESBL-producing *E. coli* and *Klebsiella* spp. as dominant MDR pathogens and urinary tract infections as a common source of bacteremia. A different study investigating this same concept in cancer patients highlights the importance of addressing the increasing prevalence of carbapenem-resistant *Enterobacteriaceae* (CRE), ESBL-producing *E. coli*, *Klebsiella pneumoniae*, *Acinetobacter baumannii*, *Pseudomonas aeruginosa*, and *Stenotrophomonas maltophilia* [9]. The findings presented in previous studies underscore the critical need for optimized empiric antibiotic therapy, rapid diagnostic tools, and antimicrobial stewardship to improve outcomes in immunocompromised cancer patients. Our study supports a call to address this increase as most of these bacteria were found in both the cancer patients and the non-cancer patients included in our study.

Although our study did not find a difference between the initiation of empirical antibiotic therapy in cancer and non-cancer patients, all measures should be taken to provide an early appropriate treatment as soon as possible in order to prevent further drug resistance and improve clinical outcomes. An increase in drug resistance as a result of the inappropriate use of antibiotics has been documented by Gudiol et al., which emphasizes the importance of correct treatment [7]. A single recent center retrospective study has demonstrated a decrease in MDR GNB infections in their hospital in the most recent data analyzed (from 2019 to 2021), which they hypothesize may be a result of reduced antibiotic exposure duration and decreased use of empirical antibacterial drugs in their institution during this period [17]. These findings highlight the critical need for continued efforts to optimize antibiotic stewardship and minimize unnecessary antibiotic use to combat the growing threat of drug resistance [18]. One factor making prompt appropriate antibiotic therapy harder to access is the potentially high cost of these strategies. A study investigating this issue stated that although the multiplex PCR system from positive blood cultures is a diagnostic strategy that shortens the identification time of the microorganism and some resistance genes, its main limitation is its high cost [19]. Although the high cost is an issue, these measures could reduce the time until effective treatment by 10 h. Strategies to decrease the cost of these methods should be consistent [20].

Additionally, the increased mortality observed with carbapenem-resistant *Klebsiella pneumoniae* and *Acinetobacter baumannii* suggests that patients infected with these organisms may require more aggressive therapeutic strategies, including combination of novel antibiotic therapy. Lin et al. observed in a multicenter retrospective study (from 2015 to 2019) that colistin-based therapy improved treatment outcomes in critically ill ICU patients with end-stage renal disease suffering from *Acinetobacter baumanni*-related BSI [21]. Our study supports finding effective treatment as quickly as possible since cancer patients had significantly higher mortality rates and were more likely to harbor MDR pathogens. This suggests that novel treatment strategies are necessary to improve survival outcomes in this vulnerable group. A study investigating CRPA bloodstream infections in patients with hematologic malignancies found that early antibiotic treatment improves survival outcomes, underscoring the importance of timely and effective antimicrobial therapy in managing resistant infections [8,14]. It should also be mentioned that understanding the local distribution patterns of pathogens and antibiotic susceptibility should be taken into consideration when dealing with these critically ill patients [22]. Risk assessment scores have been proposed to help predict the likelihood of MDR GNB infections in cancer patients in the hope of reducing the time until appropriate antibiotic treatment and improving outcomes. These scoring systems integrate factors like prior antibiotic exposure, neutropenia duration, and hospital-acquired infections, providing valuable tools for early identification and targeted intervention [23]. Our study’s findings reinforce the need for such predictive models as our data demonstrate significant associations between prior hospitalization and increased mortality in cancer patients with MDR-GNB infections.

### 4.1. Limitations

A key strength of this study is its comparative approach, providing insights into how MDR-GNB affects cancer vs. non-cancer patients differently. However, limitations include its retrospective nature, which may be impacted by the lack of longitudinal follow-up data to assess long-term outcomes. Additional limitations are the single-center nature of this study and the small sample size. Future research should incorporate prospective multicenter analyses to validate these findings and to highlight the impact of novel antibiotics treatment according to the Infectious Diseases Society of America (IDSA) guidance. 

### 4.2. Future Directions

Additional studies should explore multicenter data to determine whether these findings are generalizable across different institutions. Furthermore, genotypic studies on MDR-GNB isolates could provide insights into regional resistance patterns and guide precision-based treatment strategies. Incorporation of rapid diagnostic platforms, such as blood culture identification (BCID) PCR panels, may further facilitate the early detection of resistance determinants and enable timely the optimization of antimicrobial therapy. Together with personalized risk assessment models, these approaches may be essential in improving treatment outcomes in this vulnerable population.

## 5. Conclusions

Our study highlights the significant burden of MDR-GNB in both cancer and non-cancer patients, with notable differences in risk factors, clinical outcomes, and mortality rates. This study identified a higher 30-day mortality rate in cancer patients, emphasizing their vulnerability to MDR infections and the urgent need for targeted interventions. The predominance of Enterobacteriaceae species further underscores the necessity of optimized empiric antibiotic strategies. Additionally, the association between recent hospitalization and MDR infections reinforces the role of healthcare exposure in resistance development, suggesting a need for stricter infection control measures. These findings support the implementation of enhanced antimicrobial stewardship programs, rapid diagnostic tools, and early screening protocols to improve patient outcomes. Future research should focus on personalized treatment approaches and risk stratification to combat the growing threat of AMR in high-risk populations.

## Figures and Tables

**Table 1:** Demographics and underlying medical comorbidities.

Variable	Cancer (*n* = 31)	Non-Cancer (*n* = 81)	*p*-Value
Age	67.82 ± 11.43	55.20 ± 16.70	<0.001
Race			
Black	15 (48.4%)	59 (72.8%)	0.031
White	14 (45.2%)	21 (25.9%)	0.031
Hispanic	2 (6.5%)	1 (1.2%)	0.031
Sex			
Male	23 (74.2%)	47 (58.0%)	0.114
Female	8 (25.8%)	34 (42.0%)	0.114
Comorbidities			
Diabetes Mellitus	7 (22.6%)	39 (48.1%)	0.014
Hypertension	20 (64.5%)	52 (64.2%)	0.975
Stroke History	0 (0.0%)	18 (22.2%)	0.004
End-Stage Renal Disease	0 (0.0%)	11 (13.6%)	0.031
HIV Positive	0 (0.0%)	2 (2.5%)	0.377
COPD	4 (12.9%)	10 (12.3%)	0.936
CAD/Heart Failure	8 (25.8%)	17 (21.0%)	0.584
History of COVID-19 (past 6 months)	1 (3.2%)	13 (16.0%)	0.066

**Table 2:** Clinical characteristics and outcomes.

Variable	Cancer (*n* = 31)	Non-Cancer (*n* = 81)	*p*-Value
Infection Characteristics			
Bone Infection	1 (3.2%)	7 (8.6%)	0.319
CLABSI	2 (6.5%)	8 (9.9%)	0.570
Unknown Source	9 (29.0%)	13 (59.1%)	0.122
Heart Infection	1 (3.2%)	1 (1.2%)	0.477
Intra-abdominal Infection	2 (6.5%)	1 (1.2%)	0.126
Lung Infection	3 (9.7%)	10 (12.3%)	0.693
Genitourinary Infection	13 (41.9%)	42 (51.9%)	0.348
Skin Infection	4 (4.9%)	1 (3.2%)	0.685
*Enterobacteriaceae* spp. Isolates	26 (83.9%)	67 (82.7%)	0.884
Non-lactose fermenters	5 (16.1%)	14 (17.3%)	0.884
Quinolone Resistance	23 (74.2%)	51 (63.0%)	0.261
Clinical Symptoms			
Fever	18 (58.1%)	52 (64.2%)	0.549
Chills/Rigors	16 (51.6%)	47 (58.0%)	0.541
Clinical Outcomes			
ICU Admission	8 (25.8%)	40 (49.4%)	0.024
30-Day Mortality	12 (39.0%)	13 (16.4%)	0.017
WBC < 12,000	17 (54.8%)	28 (34.6%)	0.050
WBC > 12,000	14 (45.2%)	53 (65.4%)	0.050
Platelets < 150,000	15 (48.4%)	25 (31.6%)	0.101
Platelets > 150,000	16 (51.6%)	54 (68.4%)	0.101
Procalcitonin < 0.5	5 (20.8%)	7 (10.1%)	0.178
Procalcitonin > 0.5	19 (79.2%)	62 (89.9%)	0.178
Lactic Acid < 2	9 (29.0%)	25 (30.9%)	0.850
Lactic Acid > 2	22 (71.0%)	56 (69.1%)	0.850
Recent Hospital Admission (Last 3 Months)	21 (67.7%)	33 (40.7%)	0.011
ID Consultation	7 (22.6%)	34 (42.0%)	0.057
Hospice Admission	7 (22.6%)	3 (3.7%)	0.002
Treatment and Antibiotic Use			
Empiric Antibiotic Therapy	16 (51.6%)	52 (64.2%)	0.222
Appropriate Antibiotic Coverage Provided	21 (67.7%)	69 (85.2%)	0.038
Targeted Therapy Given	27 (87.1%)	77 (95.1%)	0.143
Days to Targeted Therapy Initiation			
1 Day	9 (33.3%)	34 (44.2%)	0.169
2–3 Days	14 (51.9%)	21 (27.3%)	0.169
4–6 Days	3 (11.1%)	18 (23.4%)	0.169
7–9 Days	0 (0.0%)	2 (2.6%)	0.169
10+ Days	1 (3.7%)	2 (2.6%)	0.169

**Table 3:** Bivariate odds ratios for key clinical variables stratified by cancer status.

**(A) Patients with Cancer (*n* = 31)**		
Variable	Odds Ratio (OR)	95% Confidence Interval
Diabetes Mellitus	2.39	1.13–5.07
ICU Admission	2.16	1.06–4.40
Recent Hospital Admission (Last 3 Months)	3.06	1.28–7.32
Hospice Admission	7.58	1.82–31.62
**(B) Patients without Cancer (*n* = 81)**		
Variable	Odds Ratio (OR)	95% Confidence Interval
Recent Hospital Admission (Last 3 Months)	1.35	1.06–1.73

**Table 4:** Multivariable logistic regression predicting 30-Day mortality (N = 112).

Predictor	Adjusted Odds Ratio (aOR)	95% CI	*p*-Value
Cancer (Yes vs. No)	3.82	1.23–11.86	0.020
Sex (Male vs. Female)	1.06	0.40–2.81	0.913
Diabetes Mellitus (Yes vs. No)	0.96	0.35–2.59	0.930
ICU Admission (Yes vs. No)	2.35	0.88–6.29	0.090
Recent Hospitalization (Yes vs. No)	0.92	0.35–2.45	0.867
Ethnicity (Non-White vs. White)	0.48	0.16–1.45	0.194
Age ≥ 40 y (vs. ≤39 y) *	1.74	0.34–8.87	0.504

* Age was categorized as ≥40 years vs. ≤39 years. Reference categories are “No,” “Female,” “White,” and “≤39 years.”

**Table 5:** Distribution of multidrug-resistant Gram-negative organisms in cancer vs. non-cancer patients.

Organism (Resistance Type)	Cancer (*n* = 31)	Non-Cancer (*n* = 81)	Total (N = 112)
*Escherichia coli (ESBL)*	19 (61.3%)	41 (50.6%)	60 (53.6%)
*Klebsiella pneumoniae (ESBL)*	3 (9.7%)	16 (19.8%)	19 (17.0%)
*Klebsiella pneumoniae (CRO)*	1 (3.2%)	2 (2.5%)	3 (2.7%)
*Klebsiella oxytoca (ESBL)*	1 (3.2%)	2 (2.5%)	3 (2.7%)
*Klebsiella oxytoca (CRO)*	0	1 (1.2%)	1 (0.9%)
*Proteus mirabilis (ESBL)*	1 (3.2%)	9 (11.1%)	10 (8.9%)
*Pseudomonas aeruginosa (CRO)*	3 (9.7%)	7 (8.6%)	10 (8.9%)
*Acinetobacter baumannii/haemolyticus (CRO)*	3 (9.7%)	8 (9.9%)	11 (9.8%)
*Serratia marcescens (CRO)*	0	1 (1.2%)	1 (0.9%)

## Data Availability

The original contributions presented in this study are included in the article. Further inquiries can be directed to the corresponding author.
